# Ten-year experience with testicular cancer at a tertiary care hospital in a resource-limited setting: a single centre experience in Tanzania

**DOI:** 10.1186/1477-7819-12-356

**Published:** 2014-11-24

**Authors:** Phillipo L Chalya, Samson Simbila, Peter F Rambau

**Affiliations:** Department of Surgery, Bugando Medical Centre, Mwanza, Tanzania; Urology Unit, Bugando Medical Centre, Mwanza, Tanzania; Department of Pathology, Catholic University of Health and Allied Sciences-Bugando, Mwanza, Tanzania

**Keywords:** Challenges, clinicopathological pattern, incidence, management, Tanzania, testicular cancers

## Abstract

**Background:**

Testicular cancers constitute major therapeutic challenges in resource-limited countries and still carry poor outcomes. There is a paucity of published data regarding testicular cancer in Tanzania, and Bugando Medical Centre in particular. This study describes the clinicopathological pattern, treatment outcome and challenges in the management of testicular cancer in our local setting.

**Methods:**

This was a retrospective study including all patients who had had histopathologically confirmed testicular cancer at Bugando Medical Centre between February 2004 and January 2014.

**Results:**

A total of 56 testicular cancer patients were enrolled in the study, representing 0.9% of all malignancies. The median age of patients at presentation was 28 years, with a peak incidence in the 21-to-30-year age group. A family history of testicular cancer was reported in four (5.4%) patients. A history of cryptorchidism was reported in six (10.7%) patients. Most patients (57.1%) presented late with an advanced stage of cancer. Testicular swelling was the main complaint in 48 (85.7%) patients. The right testis was involved in 67.9% of cases. Lymph node and distant metastases were documented in 10 (17.9%) and 12 (21.4%) patients, respectively. Histologically, 80.4% of patients had germ cell cancers, with seminoma accounting for 62.2% of cases. The most common surgical procedure was inguinal orchidectomy (77.4%). Adjuvant chemotherapy and radiotherapy were used in six (11.1%) and four (7.4%) patients, respectively. Eight (14.3%) patients died. The main predictors of mortality (*P* < 0.001) were patient’s age (>65 years), late presentation (>6 months), stage of disease, and presence of metastasis at time of diagnosis. The mean follow-up period was 22 months. At the end of five years, only 18 (37.5%) patients were available for follow-up and the overall 5-year survival rate was 22.2%. The main predictors of 5-year survival rate (*P* < 0.001) were patients’ age, stage of disease, and presence of lymph node and distant metastases.

**Conclusions:**

Testicular cancers, though rare in our setting, still carries a poor prognosis. Late presentation, poverty, paucity of resources and the high cost of newer imaging and treatment modalities are major challenges to management. Better health funding and education regarding testicular self-examination is essential.

## Background

Testicular cancer is a relatively rare tumour type, accounting for approximately 1% of all male cancers globally[1]. However, testicular cancer has a very distinctive age distribution and in many developed countries it is the most commonly diagnosed malignancy among men aged between 15 and 40 years[2]. In recent decades, the incidence of testicular cancer has been increasing, with a doubling observed since the 1960s in many Western societies. Both western and northern Europe have high age-standardized incidence rates of 7.8 and 6.7 per 100,000 men, respectively, compared with rates of 0.6 per 100,000 men in the black population of African descent[3]. However, despite these observed trends in incidence and geographical variations, few hypotheses exist to explain them[4].

Testicular cancers may appear at any age but tend to occur in three distinct age groups: infants and children (0 to 10 years), young adults (15 to 40 years) and older adults (over 60 years)[5–7]. This tumour grows rapidly with a doubling time of 20 to 30 days and has a high risk of metastatic spread[6–8].

Epidemiological risk factors for the development of testicular tumours include: a history of cryptorchidism, Klinefelter’s syndrome, a familial history of testicular tumours among first-degree relatives (father or brothers), the presence of a contralateral tumour, and infertility[8–10]. Trauma, hormones such as diethylstilboestrol, and non-specific mumps-associated testicular atrophy have also been implicated as risk factors, but there are very little data to support this[8].

Although testicular cancer can be derived from any cell type found in the testicles, more than 95% of testicular cancers arise from germ cells. The germ cell testicular cancers are classified into seminoma (classic, anaplastic and spermatocytic variants) and non-seminomatous germ cell tumours (embryonal carcinoma, teratocarcinoma, teratoma, choriocarcinoma and yolk sac tumours). Non-germ cell tumours include sex cord-gonadal stromal tumours (derived from Leydig cells or Sertoli cells) and miscellaneous neoplasms[11, 12].

Testicular cancer has become one of the most curable solid tumours and serves as a model for multimodal treatment of malignancies[13]. The treatment of testicular tumours has greatly evolved from extirpative surgery only to cisplatin-based chemotherapy, radiotherapy and retroperitoneal lymph node dissection. These methods have now rendered the condition potentially curable, and there is documentation of excellent survival figures in the developed world[13]. However, in the developing world, for example, in Tanzania, the treatment outcome is still poor[6–8, 13–15]. Reasons for this trend include a lack of well-established first-line medical treatment that leads to late diagnosis and referral, and unavailability of treatment, poverty and reluctance to accept chemotherapy and radiotherapy[8, 13, 15].

There is a paucity of information regarding testicular cancer in Tanzania and Bugando Medical Centre in particular. This is partly due to a lack of published local data regarding this condition and a lack of cancer registries in this region. This study was designed to describe our experiences of the management of testicular cancer, highlighting the clinicopathological pattern, treatment outcome and challenges in the management of testicular cancer in our local setting.

## Methods

This was a retrospective study including all patients who had histopathologically confirmed testicular cancer at Bugando Medical Centre over a 10-year period between February 2004 and January 2014.

Bugando Medical Centre is a tertiary and teaching hospital for the Catholic University of Health and Allied Sciences, Bugando in the north-western part of the United Republic of Tanzania. It is situated along the shores of Lake Victoria in Mwanza City. It has 1000 beds and serves as a referral centre for tertiary specialist care for a catchment population of approximately 13 million people. The hospital has a newly established oncology department, which provides care for all patients with histopathologically proven cancers, including testicular cancers. However, the department does not currently provide radiotherapy services, owing to a lack of this facility at our centre. As a result, patients requiring this modality of treatment have to travel long distances to receive radiotherapy at the Tanzania Tumour Centre, which is located a considerable distance from the study area.

The study population included all patients who had had histopathologically confirmed testicular cancer at our hospital during the period studied. Patients with incomplete data were excluded from the study.

The details of patients were collected from patients’ files kept in the medical records department, surgical wards, operating theatres and the histopathology laboratory. Diagnosis of testicular tumours was made on clinical grounds (palpable testicular mass with or without abdominal mass), scrotal-abdominal ultrasonography, chest radiography, intravenous urography and estimation of tumour markers (β-human chorionic gonadotrophin and α-fetoprotein). Computed tomography (CT) was not done because this facility is not available at our centre. Clinical staging was according to the Royal Marsden staging system (Table 1). All diagnoses were confirmed by histopathological analysis of the operative specimens. Relevant information regarding age at presentation, clinical presentation, investigation and stage of disease, histological type, treatment given, duration of follow-up, special management problems and outcome were collected using a preformed questionnaire.

**Table 1:** **Royal Marsden Hospital staging of testicular cancer**

Stage	Description
**I**	No evidence of metastasis
IM	Rising concentrations of serum markers with no other evidence of metastasis
**II**	Abdominal node metastases
A	<2 cm diameter
B	2 to 5 cm diameter
C	>5 cm diameter
**III**	Supra-diaphragmatic nodal metastasis
M	Mediastinal
N	Supraclavicular, cervical or axillary
O	No abdominal node metastases
ABC	Node sizes as for definition in stage II
**IV**	Extra-lymphatic metastases
Lung	
L1	<3 metastases
L2	≥3 metastases, <2 cm diameter
L3	≥3 metastases, one or more of which is >2 cm diameter

Stage IV subgroups include H+ (liver metastases), Br + (brain metastases), Bo + (bone).

### Statistical data analysis

Statistical analysis was performed using the Statistical Package for Social Sciences version 17.0 for Windows (SPSS, Chicago, IL, United States). The median (and interquartile range) and ranges were calculated for continuous variables, whereas proportions and frequency tables were used to summarize categorical variables. The chi-square (χ^2^) test was used to test for the significance of association between independent (predictor) and dependent (outcome) variables in the categorical variables. Significance was considered for *P* < 0.05. Multivariate logistic regression analysis was used to determine predictor variables that predicted the outcome.

### Ethical consideration

Ethical approval to conduct the study was obtained from the joint institutional ethic review committee of the Catholic University of Health and Allied Sciences-Bugando and Bugando Medical Centre before the commencement of the study.

## Results

**Figure 1:**
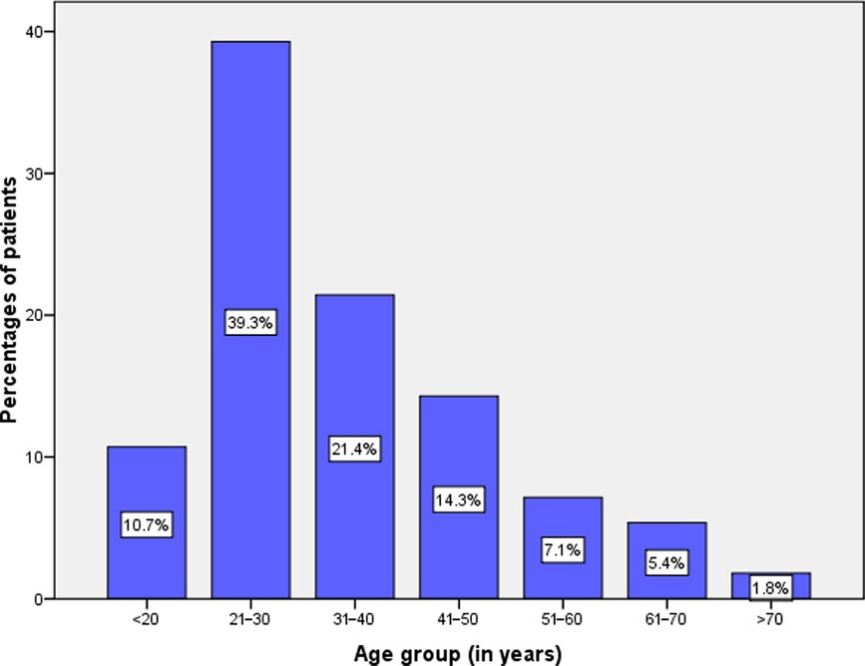
**Distribution of patients according to age group.**

During the study period, a total of 6258 malignancies were registered. Of these, 56 (0.9%) were histopathologically confirmed cases of testicular cancer; these formed the study population. Patients’ ages ranged from 15 to 72 years, with a median age of 28 years (interquartile range, 26 to 30 years). The modal age group at presentation was 21 to 30 years, accounting for 39.3% of cases (Figure 1). A total of 40 (71.4%) patients were aged 40 years and younger. A family history of testicular cancer was reported in four (5.4%) patients. A history of cryptorchidism was recorded in six (10.7%) patients. A previous history of testicular atrophy was reported in one (1.8%) patient.

The duration of symptoms at presentation ranged from 2 months to 16 months, with a median duration of 8 months (interquartile range, 6 to 10 months). The majority of patients, 32 (57.1%), presented after 6 months of the onset of symptoms, and the remaining 24 (42.9%) presented within 6 months of onset. The median time interval between onset of symptoms and presentation at our centre was significantly short in patients who presented with testicular swelling associated with pain, compared with those who presented with painless testicular swelling (*P* = 0.002). Table 2 shows the clinical presentation of the testicular cancer patients. In all, 38 (67.9%) patients had right-sided testicular cancer and 16 (28.5%) had left-sided testicular cancer. Two (3.6%) patients had bilateral testicular cancer.

**Table 2:** **Clinical presentation of testicular cancer patients** (***N*** **= 56)**

Clinical presentations	Frequency	Percentages
Testicular swelling	48	85.7
Scrotal heaviness	8	14.3
Scrotal pain	7	12.5
Abdominal swelling or mass	4	7.1
Primary infertility	2	3.6
Enlargement of breasts	2	3.6
Abnormal hair distribution	1	1.8
Jaundice	1	1.8
Decreased libido	1	1.8

Following a full history and physical examination, scrotal-abdominal ultrasonography, chest radiography and intravenous urography were done. Computed tomography was not available, which challenged our management, as this limited the staging of retroperitoneal nodes. In the absence of CT imaging, we relied on abdominal ultrasonography to detect gross retroperitoneal lymph node involvement; as a result of this, many of our patients might have been understaged, which was an obvious limitation, as staging accuracy of abdominal disease was suboptimal. Preoperative assay of tumour markers revealed elevated α-fetoprotein and β-human chorionic gonadotrophin levels in 2 (3.6%) and 16 (28.6%) patients.

Histopathologically, 82.1% of the cancers were of germ cell origin; of these, seminoma accounted for 60.9% of cases (Table 3). The majority of patients (39.3%) presented with stage IV (Table 4). Lymph node involvement was seen in 10 (17.9%) patients. Distant metastasis (Stage 4) was reported in 12 (21.4%) patients; this was mainly to the lung, liver and brain.

**Figure 2:**
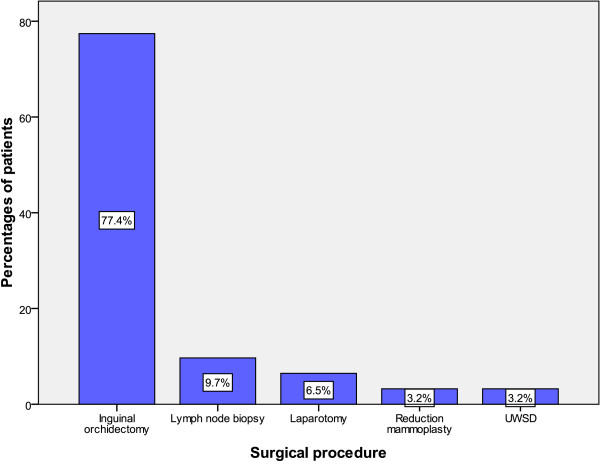
**Distribution of patients according to the surgical procedure performed.** UWSD, underwater seal drainage.

A total of 62 surgical operations were performed on 56 patients. Of these, inguinal orchidectomy was the most common surgical procedure, performed on 48 (77.4%) patients (Figure 2). Retroperitoneal lymph node dissection was not performed on any of our patients as they refused consent after being informed of the ejaculatory difficulty that might follow. Three patients (who had only incisional biopsy of the tumour) died within three days of admission and did not have any definitive treatment. Radiotherapy and chemotherapy were indicated in 54 (96.4%) patients. Of these, only 10 (17.9%) received these form of treatment modalities. Out of ten patients, six (11.1%) received chemotherapy and four (7.4%) received radiotherapy. Of those who received chemotherapy, Cisplatin-based combination chemotherapy was used in only two patients. The other four patients who had chemotherapy received a combination of actinomycin-D, adriamycin, cyclophosphamide, vincristine and vinblastin which are provided for free by the government. In addition to radical orchidectomy, one patient who had testicular lymphoma was treated with a combination of cyclophosphamide, doxorubicin, vincristine and prednisone. The other patient who had testicular adenocarcinoma from the prostate gland was treated by androgen deprivation therapy with bilateral orchidectomy and stilbestrol. The four patients who received radiotherapy had to travel long distances to receive radiotherapy at the Tanzania Tumour Centre located a considerable distance from the study area. Other patients failed to receive the recommended radiotherapy because of the prohibitive distance to the radiotherapy centres and the costs involved.

**Table 3:** **Distribution of patients according to histopathological type**

Histopathological type	Frequency	Percentages
**Germ cell tumours**	**46**	**82.1**
Seminoma	28	60.9
Embryonal carcinoma	12	26.1
Teratoma	4	8.7
Yolk sac tumour	2	4.3
**Sex cord or gonadal stromal tumours**	**4**	**7.1**
Granulosa cell tumour	2	50
Malignant Sertoli cell tumour	1	25
Leydig cell tumour	1	25
**Mixed germ cell/sex cord stromal tumours**	**4**	**7.1**
**Secondary testicular tumours**	**2**	**3.6**
Lymphoma	1	50
Adenocarcinoma from the prostate gland	1	50

**Table 4:** **Tumour stage of 56 patients with testicular cancer**

Stage	Number of patients	Percentages
I	2	3.6
II	6	10.7
III	14	25.0
IV	22	39.3
Not documented	12	21.4

Twelve postoperative complications were reported in eight patients, giving a complication rate of 14.3%. Surgical site infection was the most common post-complications, accounting for 33.3% of complications (Table 5).

**Table 5:** **Distribution of patients according to postoperative complications**

Postoperative complications	Frequency	Percentages (of 12 complications)
Surgical site infection	4	33.3
Scrotal abscess	2	16.7
Scrotal hematoma	2	16.7
Wound dehiscence	2	16.7
Fournier’s gangrene	1	8.3
Adhesive bowel obstruction	1	8.3

In this study, eight patients died in hospital, giving a mortality rate of 14.3%. According to multivariate logistic regression analysis, the patient’s age (>65 years), late presentation (>6 months), stage of disease, and presence of metastasis at the time of diagnosis were the main predictors of mortality (*P* < 0.001).

Follow-up of patients among survivors (48) ranged from 3 to 60 months with a median of 22 months (interquartile range, 18 to 24 months). At the end of the follow-up period, only 18 patients (37.5% of the survivors were available for follow-up and the remaining 30 (62.5%) patients were lost to follow-up. Out of 18 patients who were available for follow-up, only four patients were alive and well at the end of five years, giving an overall 5-year survival rate of 22.2%. According to multivariate logistic regression analysis, patient’s age at diagnosis (*P* = 0.011), stage of disease (*P* = 0.004), extent of lymph node involvement (*P* = 0.012) and distant metastasis (*P* = 0.004) were found to be independent predictors of overall survival.

## Discussion

Testicular cancers still remain relatively rare among native African men, as demonstrated in our study, where we had only 56 patients in a 10-year period, showing an incidence of 5.6 cases per year. In this study, testicular cancer accounted for 0.9% of all diagnosed malignancies seen during the study period in our setting. This concurs with figures of 0.5 to 2% that have been reported from various parts of Africa[1, 3, 4]. There is a marked variation in the incidence of testicular cancer worldwide, with Western countries having a high rate compared with Africa[6–8]. This high incidence in Western nations has been noted to be rising even further[16–18]. This has been attributed to a higher incidence of cryptorchidism, diets rich in oestrogen, increasing environmental (industrial) pollution and genetic factors[8, 19]. The low incidence observed in this study is contrary to the rising incidence in black Americans, and may be attributed to the low incidence of cryptorchidism (a major risk factor)[18, 20, 21]. Also, patients in this study were all native black Tanzanian Africans in poorly industrialized locations. Genetic studies were not done on our patients with bilateral tumours, although it is believed that many of such tumours may be hereditary in origin.

In this study, the majority of patients were in the second and third decades of life. This is in keeping with most previous reports from Africa[8, 18], but slightly at variance with reports that cancer of the testis affects young men in the third and fourth decades of life[4, 5]. It is possible that the earlier occurrence of testicular cancer in this study is related to the life expectancy in the country, rather than any special demographic feature of testicular cancer.

A family history of testicular cancer among first-degree relatives (father or brothers) has been reported to increase the risk of developing testicular cancers[8, 9, 13, 22–28]. Approximately 10% of testicular cancers appear to be genetically linked[22–25, 29]. In this study, a family history of testicular cancer was reported in 5.4% of patients, suggesting that genetic factors might play an important role in the development of this disease in Tanzania. Based on this alarming observation, we suggest that screening programmes, especially genetic screening programmes, should be considered as a main measure for prevention and control of testicular cancer in this part of the world.

The most important risk factor associated with testicular cancer is cryptorchidism. Up to 10% of all patients with testicular cancer have a history of cryptorchidism[8, 26, 30]. Epidemiologic studies have shown that patients with cryptorchidism have a 3 to 46 fold increased incidence of testicular cancer[8–10, 30]. In this study, a history of cryptorchidism was obtained in 10.7% of patients. This is comparable with the findings of others, who have reported that about 10% of all testicular cancer patients have a previous history of cryptorchidism, making it the single most important risk factor associated with testicular cancer.

In keeping with other studies in developing countries[11, 12, 22], the majority of patients (57.1%) in this study presented late with an advanced stage of cancer. Late presentation in these countries is thought to be due to ignorance, fear of consequences, long distances to hospitals, and strong beliefs in traditional medicine and faith-healers. We could not establish the reasons for late presentation in this study, owing to its retrospective nature. The late presentation of cases is an area of cancer care in our centre that requires urgent attention. Detecting primary cancer at an early stage contributes to improved chances for successful treatment and thus for survival.

As reported by other authors[12, 22], most patients with testicular cancer in this study presented with painless testicular swelling. In this study, the median time interval between the onset of symptoms and presentation to the tertiary care hospital was significantly short in patients who presented with testicular swelling associated with pain, as compared with those who presented with painless testicular swelling. A painless testicular swelling that does not interfere with normal duties and a lack of awareness of the disease might be a factor contributing to late presentation in this study, as patients with testicular swelling associated with pain are more likely to present early than those without pain. In agreement with other studies[11, 12, 22], the right testis was frequently affected in our series. Two patients in this study had bilateral testicular cancer. We could not find in the literature any reasons for this anatomical side distribution.

In this study, the diagnosis of testicular tumours was made on clinical grounds (palpable testicular mass with or without abdominal mass), scrotal-abdominal ultrasonography (to assess scrotal and abdominal masses), chest radiography (to rule out pulmonary metastases), intravenous urography and estimation of tumour markers (β-human chorionic gonadotrophin and α-fetoprotein). These tumour markers are useful in diagnosis, initial staging, assessing response to therapy and early detection of relapse in germ cell cancers[31]. Computed tomography is an important imaging tool for assessing retroperitoneal metastatic disease. In this study, CT was not done because it was not available during the study period. This challenged our management, as it limited the staging of retroperitoneal nodes. In the absence of CT, we relied on abdominal ultrasonography to detect gross retroperitoneal lymph node involvement.

Testicular cancers are known to be of germ cell origin in more than 95% of cases and constitute the most common malignancy in men aged 18 to 35 years[32]. Seminoma is reported to be the most common testicular cancer and accounts for approximately 60 to 65% of germ cell cancers[16, 21, 22, 31–33]. In this study, 80.4% of the cancers were of germ cell origin, of which seminoma accounted for 62.2% of cases. This concurs with Opot and Magoha[12] in Kenya, who reported germ cell cancers in more than 89% of cases, with seminoma accounting for 67.4% of cases, but at variance with Magoha[11] in Nigeria, who reported embryonal carcinoma as the most common germ cell cancers. Izegbu *et al.*[34] in Nigeria reported yolk sac tumours as the commonest histological subtype. We could not establish the reasons for these histopathological differences.

In this study, the majority of patients presented late with an advanced stage of cancer, which is in keeping with findings of other studies in developing countries[11, 12, 22, 33]. These findings are at variance with what is reported in developed countries, where most patients present early, at an early stage of the disease[20, 21].

Late presentation and advanced disease at the time of diagnosis in most developing countries may be explained by delay in seeking medical services, poor testicular cancer awareness, poor referral systems and medical services and a lack of screening programmes for testicular cancer.

In this study, lymph node and distant metastases at the time of diagnosis were recorded in 17.9% and 21.4% of cases, respectively. A similar metastatic pattern was recorded by Izegbu *et al.*[34] in Nigeria. Lymph node and distant metastases in this study may be underreported, owing to a lack of CT, which is an important facility in assessing retroperitoneal and mediastinal lymph nodes metastases; as a result many of our patients might have been understaged.

The treatment of testicular cancer consists of radical orchidectomy, cisplatin-based chemotherapy, radiotherapy and retroperitoneal lymph node dissection. The management of testicular cancer depends on clinical stage and histological diagnosis, although the initial treatment for most testicular tumours is orchidectomy. In this study, 48 (77.4%) patients had radical inguinal orchidectomy. The inguinal approach permits high division of the spermatic cord and early control of the vascular and lymphatic supply, as well as *en-block* removal of the testis with all its tunics. It remains the definitive procedure for pathological diagnosis and local treatment of testicular cancer[12, 22, 33]. No patient had transcrotal orchidectomy in this study, as this treatment increases the risk of spreading cancer cells into the scrotum and retroperitoneal lymph node. Dissection was not performed on any of our patients, owing to their refusal to consent after information regarding ejaculatory difficulty was given. This is a setback to effective treatment, which can be remedied by better patient education. Consent for radical orchidectomy was often given reluctantly, as patients perceived the procedure as emasculating, and a threat to manhood, sexuality and fertility.

In this study, only six (11.1%) patients received combination chemotherapy, although this was irregular in most instances and only two patients received the recommended cisplatin-based chemotherapy. The other four patients who received a combination of actinomycin-D, adriamycin, cyclophosphamide, vincristine and vinblastin defaulted and did not complete the course. This observation is in keeping with other African studies[11, 12, 22]. It is not clear why cisplastin-based chemotherapy, which is much more effective, was used in only two patients, although the high cost might have been a factor. Non-adherence to chemotherapy is a major challenge in cancer treatment, especially in resource-poor settings like ours. Reasons for non-adherence in most developing countries include financial difficulty, feeling relatively well after commencement of chemotherapy, resorting to alternative treatment and drug side effects. We could not establish the reasons for non-adherence to chemotherapy in our study, owing to its retrospective nature.

Adjuvant radiotherapy is an integral part of the management of testicular cancer. In this study, only four (7.4%) of patients requiring adjuvant radiotherapy had access to this modality of treatment. Adjuvant radiotherapy is required to reduce the risk of local recurrence following surgery[12, 22, 33]. Failure to access this modality of treatment in our patients can be explained by the fact that radiotherapy is not available at our tertiary care hospital and therefore patients requiring this form of treatment had to travel long distances to receive radiotherapy elsewhere. Because of lack of funds at the time of referral for radiotherapy in the majority of patients, less than 10% of patients were able to travel and received this form of treatment.

The prognosis of testicular cancer has remained poor in most developing countries, where most patients are already in an advanced stage of the disease at the time of diagnosis, which has been proven both in the present study and in most studies conducted in developing countries[11, 12, 22, 33]. However, when it is diagnosed and treated early, testicular cancers are curable; a 5-year survival rate of over 95% has been achieved in developed countries[20, 32]. The overall 5-year survival rate of 22.2% in our study is significantly lower than the survival rate of testicular cancer patients managed in developed countries[20, 32]. The low overall 5-year survival rate in this study can be explained by the fact that most of our patients generally seek medical attention when the disease has reached an advanced stage. Therefore, diagnosis is made when the chance of a full cure is low. The low overall 5-year survival rate in this study may also be explained by the fact that cisplastin-based chemotherapy combination was used in only two patients, probably owing to its high cost. It is hoped that these results will encourage surgeons in this locality to insist on cisplastin-based combination chemotherapy for testicular cancer, irrespective of costs. In our patients, the factors that significantly affected overall survival were age of the patient, stage of disease, lymph node, and distant metastases and histological type. The follow-up of patients in this study was generally poor as more than 60% of patients were lost to follow-up by the end of five years.

The major limitation of this study is the fact that information about some patients was incomplete, in view of the retrospective nature of the study. This might have introduced some bias in our findings. The large number of patients lost to follow-up was also a major limitation of this study, as it means that we may have underestimated the 5-year overall survival rates. Also, this study included patients who were evaluated and treated at a single institution, and so it might not reflect the whole population in this region, despite the fact that approximately 70% of oncology patients in northwestern Tanzania are managed at our centre. However, despite these limitations, findings from this study provide local data that can be utilized to improve the care of testicular cancer in our local setting. The challenges identified in the management of testicular cancer in our setting need to be addressed to deliver optimal care for these patients.

## Conclusions

Testicular cancer, although rare in Tanzania and Bugando Medical Centre in particular, poses a great challenge in the management of this disease. Late presentation, high-stage disease at presentation, poor accessibility to healthcare facilities, lack of diagnostic and staging facilities, absence of radiotherapy facilities in northwestern Tanzania, non-adherence to adjuvant therapy, high costs of newly recommended drugs, and lack of patient awareness of testicular self-examination are major challenges in the care of these patients. To address these challenges, we recommend that the public receive education regarding the importance of early presentation to health facilities and follow-up following treatment, a significant increase in health care funding, expansion of the health insurance scheme to cover cancer treatment, and improved patient education. The inclusion of teaching testicular self-examination in the primary and secondary school curriculum is also proposed as a preventive strategy. Establishment of radiotherapy services at our centre is highly recommended.

## Abbreviations

CT: Computed tomography.
